# Cytotoxic Properties of Titanocenyl Amides on Breast Cancer Cell Line MCF-7

**DOI:** 10.1155/2010/286298

**Published:** 2010-05-04

**Authors:** Li Ming Gao, Enrique Meléndez

**Affiliations:** Department of Chemistry, University of Puerto Rico, P.O. Box 9019, Mayagüez, PR 00681, USA

## Abstract

A new titanocenyl amide containing flavone as pendant group has been synthesized by reaction of titanocenyl carboxylic acid chloride and 7-Aminoflavone and structurally characterized by spectroscopic methods. This species and eight previously synthesized titanocenyl amide complexes have been tested in breast adenocarcinoma cancer cell line, MCF-7. The functionalization of titanocene dichloride with amides enhances the cytotoxic activity in MCF-7. Two sets of titanocenyl amides can be identified, with IC_50_ <100 *μ*M and IC_50_>100 *μ*M. The most cytotoxic species is Cp(CpCO-NH-C_6_H_4_-(CH_2_)_2_CH_3_)TiCl_2_ with an IC_50_ of 24(2) *μ*M, followed by Cp(CpCO-NH-C_6_H_4_-Br)TiCl_2_, IC_50_ of 46(4) *μ*M and Cp(CpCO-NH-C_6_H_4_-OCF_3_)TiCl_2_, IC_50_ of 49(6) *μ*M. There is no correlation between the nature of the para substituent on the phenyl ring and the cytotoxic properties on MCF-7 cell line.

## 1. Introduction

The development of efficient metal-based anticancer drugs currently still is a scientific challenge. The design of such species requires careful selection of the metal center and ligands surrounding their coordination sphere in order to achieve the desired biological activity but, keeping in mind that it is also desirable to maintain low-toxic side effects. In 1979, Köpf and Köpf-Maier opened a new chapter in the medicinal chemistry with the discovery of the first metallocene-based organometallic anticancer agent, titanocene dichloride, Cp_2_TiCl_2_. The fact that it possesses antitumor properties in cancer cell lines that are insensitive to cisplatin as well as lower toxic effects than cisplatin, has motivated the scientific community to continue investigating this species [1–8]. 

**Scheme 1:**
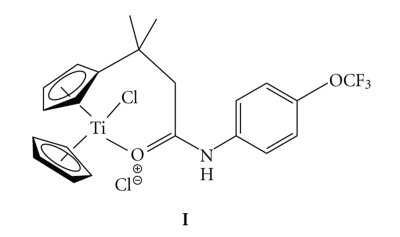


The structure modification of titanocene dichloride to enhance its anticancer properties requires a careful selection of the functional group to be appended to the cyclopentadienyl ring or replacement of the ancillary ligands for more active ones. Recently, we published the synthesis, structure, and biological activity of titanocenyl amide complexes in colon cancer cell line HT-29 [9]. We were able to achieve cytotoxic activities (IC_50_ values) on HT-29 in the micromolar range, which are two orders of magnitude more cytotoxic than titanocene dichloride as is the case for the titanocenyl amide containing a trifluoromethoxy group on the para position of the phenyl ring in Scheme 1 [9]. Apparently, the Ti-O (amide) coordination provided more stability in aqueous solution (resisting hydrolysis) and resulted in the formation of more cytotoxic species [9]. Motivated by these optimistic results, we decided to explore their activity on breast cancer cell line MCF-7. Herein we report our findings.

## 2. Experimental Details

### 2.1. General Procedure

All reactions were run under an atmosphere of dry nitrogen using Schlenk glassware or a glovebox, unless otherwise stated. Reaction vessels were flame-dried under a stream of nitrogen, and anhydrous solvents were transferred by oven-dried syringes or cannula. Tetrahydrofuran was dried and deoxygenated by distillation over K-benzophenone under nitrogen. Infrared spectra were obtained in dried KBr pellets. The NMR spectra were obtained on a DRX-500 MHz Bruker spectrometer. For the samples prepared on CDCl_3_, chemical shifts were reference relative to CHCl_3_ at 7.27 ppm (^1^H-NMR) and CHCl_3_ at 77.00 ppm (^13^CNMR) as internal standard. Analytical data were obtained from Atlantic Microlab Inc. 

The breast adenocarcinoma cell line MCF7 was purchased from American Type Culture Collection and was kept at 37°C and 95% Air/5% CO_2_. Growth medium for MCF7 was Eagle's Minimum Essential Media supplemented with 10% (v/v) fetal bovine serum, 1% (v/v) antibiotic/antimycotic, nonessential aminoacids, and 0.01 mg/mL bovine insulin. MTT and Triton X-100 used for the cytotoxic assay were obtained from Sigma. All MTT manipulations were performed in a dark room.

### 2.2. Synthesis and Characterization

*Titanocenyl carboxylic acid chloride and its precursor* were prepared as described by Gansäuer and coworkers [10, 11].

**Synthesis of Complex (9)**

Titaniumcarboxylate (0.25 mmoL, 77.4 mg) was dissolved in SOCl_2_ (1.0 mL), and stirred for 2 h at rt. Excess SOCl_2_ was removed under high-vacuum and dried for 24 h. The precipitate was dissolved in CH_2_Cl_2_ (2.0 mL), added dropwise to a mixture of the NaH (0.75 mmoL, 18 mg) and the 7-Aminoflavone (0.25 mmoL, 59.4 mg) in CH_2_Cl_2_ (6.0 mL) and stirred for another 20 h. After filtration through celite, the solvent was washed with a mixture of 1N HCl and NaCl (1.0 g each 10 mL) (2 × 5.0 mL). The organic layer was dried in MgSO_4_ and the solvent removed under reduced pressure. The crude product then chromatographed on Bio-Bead S-X3 (200–400 mesh). Before use, the biobeads were swollen in CH_2_Cl_2_ for 24 h and the product was eluted with methylene chloride to give 0.121 g (85%) of brown red solid. The product was crystallized in dichloromethane/hexane at −20°C and a brown red solid could be obtained.^ 1^HNMR (500 MHz, CDCl_3_), *δ* (ppm): 13.98 (s, 1H; NH), 8.23 (d, ^3^ *J* = 8.0 Hz, 1H; H-5), 8.08 (s, 1H; H-8), 7.96 (m, 2H; H-2′, 6′), 7.84 (d, *^3^J* = 8.0Hz, 1H; H-6), 7.56–7.54 (m, 3H; H-3′, 4′, 5′), 7.42 (m, 1H; Cp), 7.15 (m, 1H; Cp), 6.84 (s, 1H; H-3), 6.77 (s, 5H; Cp), 6.72 (m, 1H; Cp), 6.14 (m, 1H; Cp), **5.32 (CH_2_Cl_2_)**, 3.76 (d, *^2^J* = 14.0 Hz, 1H), 3.25 (d, *^2^J* = 14.0Hz, 1H), **1.70 (H_2_O),** 1.41 (s, 3H), 1.27 (s, 3H). ^13^CNMR (125 MHz, CDCl_3_), *δ*(ppm): 177.6, 177.1, 163.8, 156.4, 151.3, 139.7, 131.9, 131.3, 129.2, 126.8, 126.4, 124.9, 122.4, 121.8, 121.1, 119.3, 116.5, 111.3, 111.2, 107.5, 47.7, 35.6, 29.7, 25.7. IR (KBr, cm^−1^): 2923, 2852, 1626, 1551, 1449, 1427, 1370, 1285, 1245, 1183, 1010, 909, 829, 772. Anal. Calcd for C_30_H_27_Cl_2_NO_3_Ti∗2/3CH_2_Cl_2_∗H_2_O: C, 57.33; H, 4.76; N, 2.18. Found: C, 57.77; H, 5.10; N, 2.17.

### 2.3. Cytotoxic Assay

Biological activity was determined using the MTT assay originally described by Mossman [12] but using 10% Triton in isopropanol as a solvent for the MTT formazan crystals [13]. HT29 and MCF7 cells were maintained at 37°C and 95% Air/5% CO_2_ in McCoy's 5A (ATCC) complete medium, which had been supplemented with 10% (v/v) fetal bovine serum (ATCC) and 1% (v/v) antibiotic/antimycotic (Sigma). Asynchronously growing cells were seeded at 1.5 × 10^4^ cells per well in 96-well plates containing 100 *μ*L of complete growth medium, and allowed to recover overnight. Various concentrations of the complexes (1–1300 *μ*M) dissolved in 5% DMSO/95% Medium were added to the wells (eight wells per concentration; experiments performed in quadruplicate plates). The complexes' solutions were prepared first by dissolving the corresponding titanocenyl in DMSO and then Medium was added to a final composition of 5% DMSO/95% Medium. In addition to the cells treated with the titanocenyls, two controls experiments were run: one without any addition of solvent mixture (5% DMSO/95% Medium) and one adding 5% DMSO/95% Medium to the cells. Both control experiments behaved identical, showing that 5% of DMSO in the Medium did not have any toxic effect on the cell growth. The cells were incubated for an additional 70 hours. After this time, MTT dissolved in complete growth medium was added to each well to a final concentration of 1.0 mg/mL and incubated for two additional hours. After this period of time, all MTT containing medium was removed; cells were washed with cold PBS and dissolved with 200 *μ*L of a 10% (v/v) Triton X-100 solution in isopropanol. After complete dissolution of the formazan crystals, well absorbances were recorded in triplicates on a 340 ATTC Microplate Reader (SLT Lab Instruments) at 570 nm with background subtraction at 630 nm. Concentrations of compounds required to inhibit cell proliferation by 50% (IC_50_) were calculated by fitting data to a four-parameter logistic plot by means of SigmaPlot software from SPSS.

## 3. Results and discussion

The syntheses of eight of the nine titanocenyl amide complexes presented have been reported previously by our group [9]. We applied the synthetic methodology developed by Gansäuer and co-workers [10, 11]. A new titanocenyl amide complex, **9**, has been synthesized and spectroscopically characterized, to complete the series of titanocenyls with a wide variety of substituents on the para position of the phenyl ring (see experimental). The ^1^H NMR spectrum shows a signal at 13.98 corresponding to the NH group and four multiplets from 7.42 to 6.14 ppm attributed to the substituted Cp ring. In the ^13^C NMR spectrum shows three peaks at 177.6, 177.1, and 163.8 ppm corresponding to the three carbonyl groups. The IR spectrum corroborated the presence of the carbonyl groups with a band at 1626 cm^−1^.

The cytotoxicities of the titanocenyl complexes on breast adenocarcinoma cancer MCF-7 cell line were measured using a slightly modified MTT assay at 72 hours [12, 13]. As a reference, the cytotoxic activity of Cp_2_TiCl_2_ was tested at 72 hours and an IC_50_ value of 570(5) *μ*M was obtained. In addition, two control experiments were run in 100% Medium and 5% DMSO/95% Medium. Both control experiments behaved identically, demonstrating that 5% DMSO in the Medium does not have any cytotoxic effect on these cells. 

**Figure 1:**
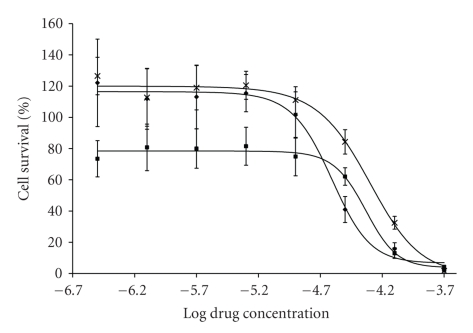
Dose-response curves for selected Amide-Functionalized Titanocenyls complexes against MCF-7 breast cancer cells at 72 hours of drug exposure. Legend: complex-**1** (squares), complex-**6** (asterisks), complex-**7** (diamonds). Experiments run in quadruplicates.

The objective of this study is to investigate the role of the substituents on the phenyl ring with different polarities, steric and electrodonating capabilities and the resulting anticancer properties on breast cancer. Figure 1 depicts the dose-response curve for the most active titanocenyls and Table 1 summarizes the results of the in vitro cytotoxicity experiments on MCF-7 breast cancer cell line as determined by MTT assay. The IC_50_ value represents the concentration of the titanocenyl at which the cell growth is inhibited by 50%. 

Upon analysis of Table 1, it can be noted that all the functionalized titanocenyls are more cytotoxic than Cp_2_TiCl_2_ (IC_50_ = 570(5) *μ*M). Identical pattern was observed for these species on HT-29 colon cancer cell line (see Comparative Table in Supplementary Material) [9]. As previously reported, the amide functionalization increases the cytotoxic activity of the titanocenes as compared to Cp_2_TiCl_2_ [9]. Second, the titanocenyls fall in two categories: highly cytotoxic species with IC_50_ < 100 *μ*M and moderately cyctotoxic species with IC_50_> 100 *μ*M. The most cytotoxic species is Cp(CpCO-NH-C_6_H_4_-(CH_2_)_2_CH_3_)TiCl_2_ with an IC_50_ of 24(2) *μ*M, followed by Cp(CpCO-NH-C_6_H_4_-Br)TiCl_2_, IC_50_ of 46(4) *μ*M and Cp(CpCO-NH-C_6_H_4_-OCF_3_)TiCl_2_, IC_50_ of 49(6) *μ*M. Interestingly, these three species are the most cytotoxic in HT-29 colon cancer cell line [9]. Third, there is no correlation between para substituent on the phenyl ring (polarity, steric, and electrodonation) and cytotoxicity. Furthermore, although the titanoceneyl-flavone derivative, 9, showed an IC_50_ < 100 *μ*M, we were expecting better cytotoxic activity based on the fact that flavones have antioxidant and anticancer properties as well as serving as transport agent for drugs without side effects [14]. 

To put in perspective these titanocenyl amides, we should compare them with other functionalized titanocenes. Recently, other amide-functionlized titanocenes have been reported with anticancer properties with cytotoxicities in the 10^−5^ M range in six cancer cell lines: BJAB (lymphoma), MelHo and A375 (melanoma), MCF-7 (breast carcinoma), and Nalm-6 and Jurkat (leukemia) [15]. As it can be seen, our most active titanocenyl amides have cytotoxicities in MCF-7 similar to those reported by Gansäuer and co-workers. However, their cytotoxic data was obtained by measuring apoptosis (AC_50_) and not IC_50_ and these results must be looked carefully since the AC_50_ and not IC_50_ are determined differently. In any event, both titanocenyl amides species, those prepared by Gansäuer and co-workers and by our group, have very good response in breast cancer, MCF-7, and we believe that our species could have applications in other cancer cell lines.

Other types of functionalized titanocenes have been reported with enhanced cytotoxic properties and IC_50_ values in the micromolar range [16–22]. For instance, McGowan and co-workers reported the anticancer activity of a titanocene containing a cyclic amino pendant group attached to the cyclopentadienyl ring, [C_5_H_4_(CH_2_)_2_N-(CH_2_)_5_]_2_TiCl_2_ · 2HCl, on MCF-7 with an IC_50_ of 62 *μ*M [16]. Our more cytotoxic species, Cp(CpCO-NH-C_6_H_4_-(CH_2_)_2_CH_3_)TiCl_2_, Cp(CpCO-NH-C_6_H_4_-Br)TiCl_2_ and Cp(CpCO-NH-C_6_H_4_-OCF_3_)TiCl_2_, have IC_50_ of value below 50 *μ*M demonstrating higher cytotoxic activity than this water soluble [C_5_H_4_(CH_2_)_2_N-(CH_2_)_5_TiCl_2_ · 2HCl]. 

Beckhove and co-workers have reported anticancer properties of bis-[p-methoxybenzyl)cyclopentadienyl] titanium dichloride (Titanocene Y). This is active against a wide variety of cancer cell lines. Moreover, Titanocene Y has been tested in explanted human breast tumor cells and in xenografted MCF-7 tumors in mice with promising results in terms of doses, low toxicity, and reduction of tumor volume [21]. Also it has been tested in MCF-7 incubated in presence of serum albumin and showed good cytotoxic activity at micromolar concentrations [22]. Lately, fluorinated derivatives of Titanocene Y have been synthesized and their cytotoxic properties have been examined in Caki-1 and LLC-PK cell lines [23]. They have evidence that incorporation of fluorine on the benzyl group improves substantially its cytotoxicity when compared to the parent compound, Titanocene Y. In particular, the trifluoromethoxy group on the para position has demonstrated to increase the cytotoxic of the corresponding titanocenes as compared to the parent ones. While we have found similar evidence for our titanocenyl amides in colon cancer, HT-29 cell line, such correlation for MCF-7 cell line cannot be extrapolated. In our case, the titanocenyl with the less polar and more hydrophobic substituent (complex **7**), with a propyl group on the para position of the phenyl ring, exhibited the highest cytotoxic activity. Finally, other strategies are currently being pursued such as replacement of chlorides by fluorides as ancillary ligands on Titanocene Y and derivatives [24]. It has been found that due to the increased hydrolytic stability in the Ti–F bond, these species improved 4–7 times their cytotoxic activities in Hela and Hep cell lines when compared to the chlorides derivatives, but there are no cytotoxic studies on MCF-7 cell line on these complexes to compare with our complexes [24].

## 4. Conclusion

The functionalization of titanocene dichloride with amides (phenyl amides) increases the cytotoxic activity of the resulting titanocenyl amide complexes. One possible explanation could be the Ti-O(amide) bond which provides stability in aqueous environment and makes the complex more resistant to hydrolysis. Although this is highly speculative, based on the structural features of these titanocenyl amides and the hypothesis that albumin is the carrier protein of titanocene into the target place inside the cell [22, 25], we can envision that the phenyl ring could provide the hydrophobic environment to incorporate the titanocenyl into the hydrophobic cavities where the fatty acid docks and N–H moiety could be involved in hydrogen bonding with nearby amino acids. Another scenario could be that these cationic species are uptaken by the cells by mean of organic cation transporters [26]. Mechanistic studies and cytotoxicity of these complexes in other cell lines will be investigated in the future.

## Supplementary Material

Comparative Table of Titanocenyl Amides Complexes Between Colon Cancer HT-29 and Breast Cancer MCF-7 Cell Lines.

## Figures and Tables

**Table 1:** Cytotoxicities of titanocenyl amides studied on MCF-7 breast cancer cell line at 72 h, as determined by MTT assay. IC values are the average of four independent measurements with their standard deviations ( ).

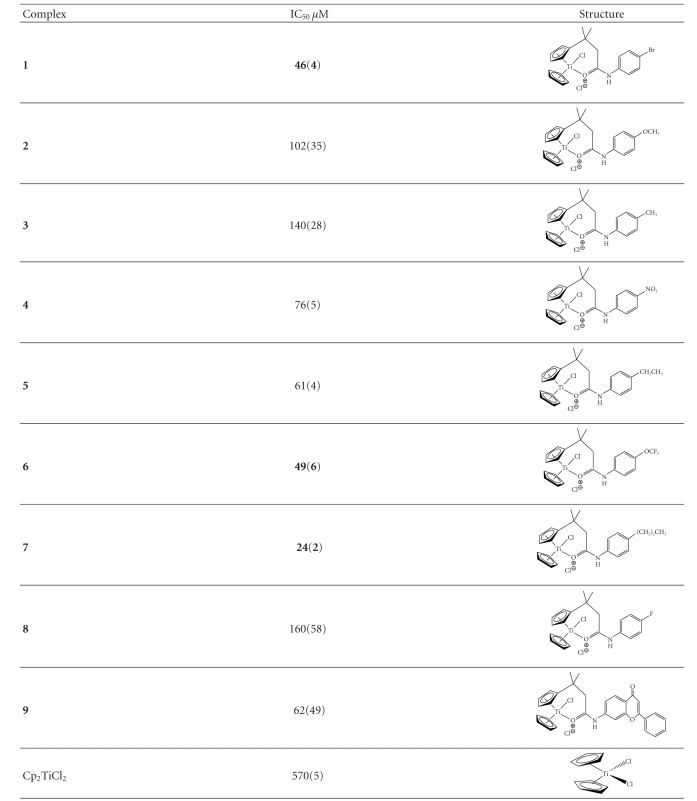

## References

[1] Köpf H, Köpf-Maier P (1979). Titanocene dichloride-the first metallocene with cancerostatic activity. *Angewandte Chemie International Edition*.

[2] Köpf-Maier P (1994). Complexes of metals other than platinum as antitumour agents. *European Journal of Clinical Pharmacology*.

[3] Köpf-Maier P, Köpf H, Fricker SP (1994). Organometallic titanium, vanadium, niobium, molybdenum and rhenium complexes early transition metal antitumor drugs. *Metal Compounds in Cancer Therapy*.

[4] Harding MM, Mokdsi G (2000). Antitumour metallocenes: structure-activity studies and interactions with biomolecules. *Current Medicinal Chemistry*.

[5] Meléndez E (2002). Titanium complexes in cancer treatment. *Critical Reviews in Oncology/Hematology*.

[6] Caruso F, Rossi M (2004). Antitumor titanium compounds. *Mini-Reviews in Medicinal Chemistry*.

[7] Caruso F, Rossi M (2004). Antitumor titanium compounds and related metallocenes. *Metal Ions in Biological Systems*.

[8] Kostova I (2009). Titanium and vanadium complexes as anticancer agents. *Anti-Cancer Agents in Medicinal Chemistry*.

[9] Gao LM, Matta J, Rheingold AL, Meléndez E (2009). Synthesis, structure and biological activity of amide-functionalized titanocenyls: improving their cytotoxic properties. *Journal of Organometallic Chemistry*.

[10] Gansäuer A, Franke D, Lauterbach T, Nieger M (2005). A modular and efficient synthesis of functional Titanocenes. *Journal of the American Chemical Society*.

[11] Gansäuer A, Winkler I, Worgull D (2008). Modular synthesis of functional Titanocenes. *Organometallics*.

[12] Mossman T (1983). Rapid colorimetric assay for cellular growth and survival: application to proliferation and cytotoxycity assays. *Journal of Immunological Methods*.

[13] Denizot F, Lang R (1986). Rapid colorimetric assay for cell growth and survival—modifications to the tetrazolium dye procedure giving improved sensitivity and reliability. *Journal of Immunological Methods*.

[14] Si D, Wang X, Zhou Y-H (2009). Mechanism of CYP2C9 inhibition by flavones and flavonols. *Drug Metabolism and Disposition*.

[15] Gansäuer A, Winkler I, Worgull D, Lauterbach T, FraWagner L, Prokop A (2008). Carbonyl-substituted Titanocenes: a novel class of cytotoxic compounds with high antitumor and antileukemic activity. *Chemistry—A European Journal*.

[16] Allen OR, Croll L, Gott AL, Knox RJ, McGowan PC (2004). Functionalized cyclopentadienyl titanium organometallic compounds as new antitumor drugs. *Organometallics*.

[17] Weber H, Claffey J, Hogan M, Pampillón C, Tacke M (2008). Analyses of Titanocenes in the spheroid-based cellular angiogenesis assay. *Toxicology in Vitro*.

[18] Oberschmidt O, Hanauske AR, Pampillón C, Sweeney NJ, Strohfeldt K, Tacke M (2007). Antiproliferative activity of titanocene Y against tumor colony-forming units. *Anti-Cancer Drugs*.

[19] Pampillón C, Sweeney NJ, Strohfeldt K, Tacke M (2007). Synthesis and cytotoxicity studies of new dimethylamino-functionalised and heteroaryl-substituted titanocene anti-cancer drugs. *Journal of Organometallic Chemistry*.

[20] O’Connor K, Gill C, Tacke M (2006). Novel titanocene anti-cancer drugs and their effect on apoptosis and the apoptotic pathway in prostate cancer cells. *Apoptosis*.

[21] Beckhove P, Oberschmidt O, Hanauske AR (2007). Antitumor activity of Titanocene Y against freshly explanted human breast tumor cells and in xenografted MCF-7 tumors in mice. *Anti-Cancer Drugs*.

[22] Vessières A, Plamont M-A, Cabestaing C (2009). Proliferative and anti-proliferative effects of titanium- and iron-based metallocene anti-cancer drugs. *Journal of Organometallic Chemistry*.

[23] Claffey J, Gleeson B, Hogan M, Müller-Bunz H, Wallis D, Tacke M (2008). Fluorinated derivatives of titanocene Y: synthesis and cytotoxicity studies. *European Journal of Inorganic Chemistry*.

[24] Eger S, Immel TA, Claffey J (2010). Titanocene difluorides with improved cytotoxic activity. *Inorganic Chemistry*.

[25] Ravera M, Gabano E, Baracco S, Osella D (2009). Electrochemical evaluation of the interaction between antitumoral titanocene dichloride and biomolecules. *Inorganica Chimica Acta*.

[26] Kapp T, Müller S, Gust R (2006). Dinuclear alkylamine platinum(II) complexes of [1,2-bis(4-fluorophenyl) ethylenediamine]-platinum(II): influence of endocytosis and copper and organic cation transport systems on cellular uptake. *ChemMedChem*.

