# Supplementary material for: Cytotoxic Properties of Titanocenyl Amides on Breast Cancer Cell Line MCF-7
Source: Met Based Drugs. 2010 May 4;2010:286298. doi: 10.1155/2010/286298 (PMC2863081; doi:10.1155/2010/286298)
Supplement: Supplementary file 1 — Comparative Table of Titanocenyl Amides Complexes Between Colon Cancer HT-29 and Breast Cancer MCF-7 Cell Lines. [file 286298.f1.pdf]

Supplementary Material

Complex	IC_{50} µM HT-29	IC_{50} µM MCF-7	Structure
1	**11(2)**	**46(4)**	**R** = Br
2	**143(5)**	**102(35)**	R = OCH_3
3	**109(3)**	**140(28)**	R = CH_3
4	**23.4(1)**	**76(5)**	R = NO_2
5	**152(6)**	**61(4)**	R = CH_2CH_3
**6**	**8.9(4)**	**49(6)**	**R =** OCF_3
7	**12.8(3)**	**24(2)**	**R** = $(CH_2)_2CH_3$
8	**137.0(5)**	**160(58)**	R = F
9		**62(49)**	R= 7-Aminoflavone
Cp_2TiCl_2	**413.0(2)**	**570(5)**	
